# The Effects of Technology Entrepreneurship on Customers and Society: A Case Study of a Spanish Pharmaceutical Distribution Company

**DOI:** 10.3389/fpsyg.2016.00978

**Published:** 2016-06-28

**Authors:** Rosa M. Muñoz, Jesús D. Sánchez de Pablo, Isidro Peña, Yolanda Salinero

**Affiliations:** Business Management Department, Castilla-La Mancha UniversityCiudad Real, Spain

**Keywords:** corporate entrepreneurship, technology entrepreneurship, case study, pharmaceutical distribution, effects on customers and society

## Abstract

The main purpose of this paper is to provide an understanding, within the field of corporate entrepreneurship, of the various factors that enable technology entrepreneurship in established firms and its principal effects on customers and society. The paper reports on a case study regarding technology entrepreneurship in a Spanish company whose activity is pharmaceutical distribution. This company has been able to overcome the consequences of the worldwide crisis and start an innovative process which includes the installation of new information technology (IT) and an investment of 6 million Euros. It is, in this respect, a model to imitate and the objective of this paper is therefore to discover the managers’ entrepreneurial orientation (EO) characteristics which have made this possible, along with the organizational and social effects resulting from the process. We verify that EO is present in this company and that the development of new IT has important effects on customers and the population.

## Introduction

Entrepreneurial behavior is one of the key strategies of organizations that are seeking to acquire and sustain a competitive advantage in global markets. Researchers have coined various terms to describe this issue at the firm level: intrapreneuring, intrapreneurship, intracorporate entrepreneurship, corporate venturing, internal corporate entrepreneurship, and entrepreneurial strategy (Antoncic and Hisrich, 2004). However, for the purpose of this study, the general term corporate entrepreneurship (CE) will be used.

Although many companies state that the entrepreneurial spirit is part of their organizational cultures, it is not common to see organizations that have gained a competitive edge through the use of solid CE strategies (Demirci, 2013). CE can be defined as “*a multidimensional process with many forces acting in concert that lead to the implementation of an innovative idea*” (Hornsby et al., 1993, p. 30).

This process is initiated by the top management and includes a redefinition of the business concept, reorganization, and the introduction of system-wide changes for innovation (Tajeddini and Mueller, 2012). CE encompasses innovativeness, risk taking, and proactiveness (Zahra and Covin, 1995) and is an important determinant of firm, regional, and national economic performance (Gupta et al., 2004; Wiklund and Shepherd, 2005). Bearing these characteristics in mind, the introduction of new information technology (IT) can be considered as a part of a company’s CE. That is, opportunities to use new products and processes which stem from the development of new technology and/or the commercialization of technologies developed by others.

The need to pursue CE has arisen as the result of a variety of pressing problems including: required changes, innovations and improvements in the marketplace to avoid decline, perceived weaknesses in the traditional methods of corporate management and the turnover of innovative-minded employees who are disenchanted with bureaucratic organizations (Kuratko et al., 1990). Entrepreneurship is not limited to the start-up domain and a parallel strand in literature has been developed that stresses the importance of entrepreneurship for and within existing corporations. Entrepreneurial activities in existing organizations result in two possible types of corporate activities: strategic renewal- changes in organizational business processes and new business venturing which is related to the creation of new business units (Gómez et al., 2011).

The focus of this study is to explore the supporting factors in IT entrepreneurship and its consequences. The paper reports on a case study regarding technology entrepreneurship in a Spanish company whose activity is pharmaceutical distribution. It is a mature firm which, during the course of the last few years, has been able to adopt IT in innovative ways thanks to the entrepreneurial spirit of its managers. This rapid and necessary adaptation to the requirements of the new economy is positioning the company within its sector, and it is now one of the leaders. The firm has been able to overcome the consequences of the worldwide crisis and continue its innovative process, which includes the installation of new IT and an investment of 6 million Euros. It is, in this respect, a model to imitate, and the objective of this paper is therefore to discover the managers’ entrepreneurial orientation (EO) characteristics which have made this possible, along with the effects on the organization, customers and society resulting from the process. The research question of this study is, therefore, to investigate “how” IT entrepreneurship occurs in the company and “what” factors enable it.

Three concepts are linked throughout the paper: CE, technology entrepreneurship and IT. Schumpeter (1942) defined the entrepreneur as a person who introduces new technologies into the production process. This author argued that entrepreneurship boosts innovation, the introduction of new products, or processes. With regard to IT, this factor favors competitiveness and innovation. IT is therefore an important part of technology entrepreneurship and technology entrepreneurship is an important part of CE.

This paper is organized as follows. Section “Corporate Entrepreneurship and Information technology entrepreneurship: the influence of consumer behavior and other factors” draw on literature in the field. Section “Methodology” presents the methodology. Section “Case Study” describes the history of the organization, its main characteristics and its type of business, explaining the technology entrepreneurship of the company and paying special attention to its organizational, strategic and social implications. Finally, Section “Conclusion” presents the main conclusion and implications extracted from the case study.

## Corporate Entrepreneurship

The ideas behind CE can be traced back to the 70s, but it was not until the appearance Pinchott’s (1985) book that it became a separate topic (Christensen, 2005). Entrepreneurship is considered to be a vital component in the process of economic growth and development. Organizational performance, growth and development may depend on entrepreneurship to a considerable extent (Antoncic and Antoncic, 2011).

This phenomenon can be studied from an individual perspective by analyzing the characteristics and functions of the individual entrepreneur (Bygrave and Hofer, 1991), differences between individual entrepreneurs and non-entrepreneurs (Gartner, 1990), or the collective process of the discovery, evaluation and exploitation of opportunities (Shane and Venkataraman, 2000).

Entrepreneurship is a process that consists of revitalizing existing companies, revenue growth, profitability enhancement and pioneering the development of new products, services and processes (Bailetti, 2002; Miles and Covin, 2002). We can reflect upon the definition proposed by Davidsson (2005), which states that it: “*is about the processes of discovery and exploitation of opportunities to create future goods and services.*” A significant proportion of innovations emerge from within existing organizations (Hayton et al., 2013). Established organizations possess significant resource advantages over new start-ups: capabilities for the production, distribution and marketing of their services and products and legitimacy in their strategic fields and among stakeholders, particularly potential customers and suppliers (Miller, 1983; Rauch et al., 2009).

Innovation is at the heart of the entrepreneurial spirit (Lumpkin and Dess, 2001). Covin and Miles (1999) define innovation as “*the introduction of a new product, process, technology, system, technique, resource or capability to the firm or its markets.*” This is conceptualized as new products or processes that significantly improve customer benefit and technological delivery over existing products (Chandy and Tellis, 2000). Durand (2004) considers innovation as a process which “*goes beyond the limits of technologies to address the larger scope of change in general. Innovation can indeed deal with the technological side of human activities, thus with product design and manufacturing processes, but it may also deal with the organizational and social side, e.g., external interactions with suppliers, clients or partners, internal processes which became routines in the way the firm operates.*” In this case, we shall pay particular attention to external interactions and organizational and social effects.

Castrogiovanni et al. (2011), found that the creation of personal relationships and the development of an open communication between owner-managers and employees, and among employees themselves, can help to explain the dynamics of entrepreneurial behaviors within small firms. Openness in communication is important as regards both promoting CE activities and creating the most appropriate work environment in which to carry out other resource management practices that stimulate entrepreneurial behaviors (DeNisi, 2015).

Entrepreneurial activities carried out by the enterprise to sustain or improve its competitive position have several consequences as regards processes, structures and capabilities (Srivastava and Agrawal, 2010; Ozdemir et al., 2016). Given the importance of CE, various scholars have focused on identifying the factors that contributing to or enhance CE (see **Table 1**).

**Table 1:** Factors that influence corporate entrepreneurship.

Factors enhancing corporate entrepreneurship	Authors
*Environmental factors*: reward and motivation, management support, resource availability, team spirit, empowerment, organizational structure, and risk taking	Srivastava and Agrawal, 2010
*Characteristics of entrepreneur and employees:* risk-taking propensity, achievement motivation, energy level, need for autonomy, need for achievement, dominance, persistence, desire for personal control and the desire to build something of one’s own	
*Entrepreneur knowledge about consumer behavior*: consumers will rate the innovative attributes of a dominant brand as more important when the dominant brand introduces a core innovation as opposed to a peripheral innovation. This will not occur when a non-dominant brand innovates	Bagga et al., 2016
Management support for CE, reward and reinforcement, work discretion, time availability and organizational boundaries	Hornsby et al., 1993
Proactiveness, risk taking, and innovativeness	McDougall and Oviatt, 2000
Appropriate reward, management support, a supportive organizational structure, and belief in risk-taking and failure tolerance	Christopher et al., 2008
*Internal*: knowledge, networks, and identify business opportunities	Urbano and Turró, 2013
*External:* having fear of failure, media impact and the number of procedures needed to create a company	
*Internal*: top management values and philosophies, organizational resources and competencies, and organizational structure	Chang, 2000
*External:* external environment, the industry’s life cycle and the type of government intervention	

*Source: Authors.*

Finally, Lumpkin and Dess (1996) define EO as an organizational decision-making proclivity that favors entrepreneurial activities. There is an assumption that EO represents a continuous variable in which all organizations can be positioned. Covin and Wales (2012) explore how the concept has been portrayed and assessed in prior research. They claim that researchers are free to choose whichever measurement approach best serves their research purposes. In this respect, some authors have conceived of EO as a construct composed of three sub-dimensions: innovativeness, risk taking, and proactiveness (Miller, 1983; Covin and Slevin, 1989). Some other authors have later expanded the number of dimensions that characterize EO by adding autonomy (Dess and Lumpkin, 2001; Hughes and Morgan, 2007), and they consider that this dimension is an important characteristic of EO. The level of autonomy that managers give to employees can drive the innovations, creativity and changes usually linked to EO.

## Information Technology Entrepreneurship: The Influence of Consumer Behavior and Other Factors

Within the field of CE, we shall pay attention to the process of discovering and applying new IT systems. Scholars began to analyze the creation of technology-based firms from the 1990s, since these firms contribute to job creation and play a crucial role in renewing the economic system. Scholars have adopted diverse conceptualizations of IT, and have extended it beyond hardware and software to include a range of contextual factors associated with its application within organizations.

Information technology has been one of the most important drivers of economic and social value in the last 50 years, transforming organizations, markets, industries, societies, and the lives of individuals (Lucas et al., 2013). Understanding the economic impact of IT is a critical issue for researchers, and there is a rich body of literature concerning IT value (e.g., Wade and Hulland, 2004). Many papers have stressed the strategic significance of IT as regards supporting competitive strategies and improving firm performance (Powell and Micallef, 1997; Kohli and Devaraj, 2003; Melville et al., 2004; Pavlou and Ei Sawy, 2006; Chae et al., 2014). Others have stressed the IT-Productivity relationship at national economy level (Dedrick et al., 2013). These works are based on several theoretical paradigms, including microeconomics, industrial organization theory, and sociological and socio-political paradigms showing the complex problem of linking IT to organizational performance (Melville et al., 2004).

For many firms, the most common reasons for adopting IT are to provide a means to enhance survival and growth, thus staying competitive and enhancing innovation abilities (Nguyen, 2009). IT can add value to an organization via the functionality, usability and information structure, which in turn affect the quality, efficiency and innovations of IT users (Gustafsson et al., 2009). Adopting new IT is also a means to enhance the way in which people capture and distribute information, lower production and labor costs, add value to products and services and increase the company’s competitive advantage (Nguyen, 2009). These IT systems are widely applied in business such that not only production but also administration processes can be technology-intensive.

Information technology systems require quality interactive interfaces and compatible dynamic knowledge systems. If they are not compatible with these systems, then either the systems should be improved or replaced, or people should be better trained or replaced (Wang, 2003). If the cost of hiring new staff is high, firms must provide the current staff with training. Training and effective communication must be provided for existing employees if there is to be a substantial change in the IT (Nguyen, 2009).

Information technology systems can reduce and automate repetitive works, and reduce the time needed to search for copy, collect, and format information, and they can enable team members to focus on critical and inventive activities (Wang, 2003). Moreover, innovations in one area have important implications in other areas, and distribution impacts on the concepts of product and service (Franklin et al., 2013).

Information technology influences the organizational structure via the company’s organizational design parameters (Mintzberg, 1979): (a) job design, thereby reducing specialization since IT assumes the routine tasks. This increases autonomy within the organization. When employees have the capacity to make decisions regarding their work, this is directly related to their attitudes toward engaging in entrepreneurial activities; (b) design of the superstructure, thus cutting down the number of hierarchical levels since IT simplifies communication, coordination and control functions and therefore increases the degree of decision making authority in the possession of individuals; (c) design of the lateral linkages, thereby improving analytical and design capabilities, increasing access to information and making it easy to access the results; and (d) design of the decision-making system, thus allowing organizations to simultaneously exploit the advantages of both centralization and decentralization. In this respect, Lau et al. (2001) prove that IT has significant impacts on: (a) formalization, moving organizations toward less formalized network structures; (b) specialization, thus facilitating outsourcing process; (c) promoting the use of lateral communication; (d) team work, as this is basic to a flatter organization; and (e) the learning of organization based on open communication, coordination improvements, and training.

## Methodology

The paper adopts an exploratory perspective and employs a qualitative approach. A case study was used to gain deeper insights into a contemporary and complex issue within its real-life context (Yin, 1994). Many studies are based on qualitative methodologies and can reveal the reality of entrepreneurship in organizations (e.g., Brennan and McGowan, 2006; Gómez et al., 2011) and concretely the implantation of IT in pharmaceutical distribution (Clemons and Row, 1988; Bruque et al., 2004).

Clemons and Row (1988) analyzed McKesson’s order entry and distribution system, Economost. This system allows almost 100% of McKesson’s orders to be entered electronically by customers. The impact of Economost on McKesson’s system has been favorable, affecting: (a) the competitive position, including its profitability and market share relative to its competitors; (b) the industry as a whole, including profitability and concentration; (c) suppliers; and (d) customers, because sales personnel are no longer principally order takers and they can be used actively, as business consultants to the retailers.

Bruque et al. (2004) carried out an analysis of 16 cases in the pharmaceutical distribution sector in Spain. The results indicate frank and fluid communication between departments and members of the organization, low levels of conflict and the explicit support of top management as regards the introduction and development of IT.

The single case setting limits the applicability of the research to other institutions. However, the framework and model that are developed, along with the overall approach, are valuable contributions to an important and emerging research area. Case-based research aims to generate refined theory based on an in-depth understanding of a particular context. According to Yin (1994), research must identify some situations in which all research strategies might be relevant. The “how” and “what” questions are asked about a contemporary set of events over which the investigator has little or no control. This study investigates “how” EO occurs in the company and “what” factors enable it. As the paper seeks to address research questions, this suggests the adoption of an exploratory approach (Yin, 1994). The identification of EO enablers is essentially exploratory, in the sense that the main objective is to refine a research idea in order to facilitate further research (Kervin, 1992).

The qualitative approach and exploratory nature of the research question influenced the data-collection method. Research conducted within the qualitative paradigm is characterized by its commitment to collecting data from the context in which social phenomena naturally occur and to generating an understanding that is grounded on the perspectives of research participants (Marshall and Rossman, 1995). Data collection was consequently developed using desk and field research. Desk research, based on internal company documents, served to provide a detailed understanding of the innovation. Moreover, the field research included in-depth semi-structured interviews with the company’s Managing Director, Financial Director and IT professionals. Interviewing multiple respondents in the company provided diverse sources of evidence and served as a means to validate and replicate the findings. The interviews took place during June–July 2013.

Bearing in mind the principal opinions of the scholars reviewed above, we have included the following dimensions in our research: EO, organizational effects, company strategy and social effects. The Covin and Slevin (1989) and the Hughes and Morgan (2007) scales were chosen in order to analyze the EO in our case study. The first scale considers that EO is a composite of risk taking, innovativeness, and participativeness. We have also introduced the autonomy items from the latter because, like Hughes and Morgan, we consider the fact that the employees are permitted to act and think without interference and perform jobs that allow them to make and instigate changes to be important for EO. Furthermore, we analyze the organizational effects based on Mintzberg (1979). We have also added more questions in relation to organizational effects, company strategy and customers and social effects in order to include some important issues that affect this specific case (see **Appendix**).

First, in the following section, we describe the company, its activity and its most recent innovations. We then explain the principal ideas extracted from the interviews.

## Case Study

### The Company and the Pharmaceutical Distribution

The principal activity of the firm under analysis is that of pharmaceutical distribution. The pharmaceutical industry in Europe is defined as being composed of commercial enterprises engaged in the design, creation and development of medicines to prevent or cure disease and relieve human suffering. Distribution can be defined as a variety of processes linked with delivering the pharmaceutical products needed to the right place at the right time (such as express delivery to hospitals or daily shipments to pharmacies).

One of the most important characteristics of pharmaceutical distribution in Spain is its wide public regulation. Customers can access medicines in three main ways, which implies, respectively, 1, 21, and 78%. The national health programs fall into the first of these, which, through specific programs (concerning, for example, vaccinations), supply medicines directly to users. Hospitals make up the second, through the medication administered to hospitalized patients. The third is direct purchase by the general public, and it is in this process that the wholesalers (like Cofarcir) and the retailers (the pharmacies) participate.

**FIGURE 1:**
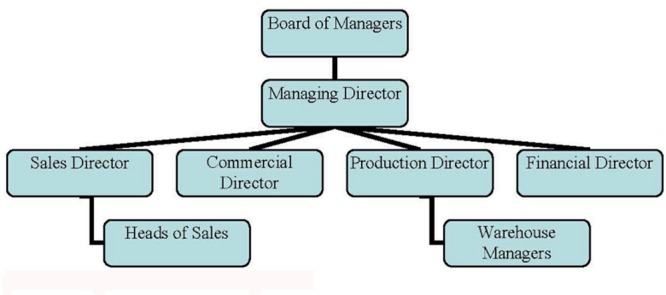
**Organization chart.** *Source: Cofarcir’s Annual Reports*.

Cooperativa Farmacéutica de Ciudad Real (Cofarcir) is a Spanish cooperative located in the city of Ciudad Real (Spain; it also has another warehouse in Alcázar de San Juan, a town in the province of Ciudad Real), whose business is the acquisition and distribution of pharmaceutical specialties for exclusive use by its partners (the members of the cooperative society) and, in general terms, every product related to the practice of the pharmaceutical profession. Its organization chart is shown in **Figure 1**.

The company started its activity in 1931, and during the course of the last 84 years of its existence has been numbered amongst the six largest companies in the province and the twenty largest companies in the country. Some data related to the company is shown in **Table 2**.

**Table 2:** Data of Cofarcir.

Variable	2013	2014
Turnover (mill. €)	101	100
Number of employees	86	86
ROA (%)	0,29	0,91
ROE (%)	1,23	4,06
Liquidity ratio	1,11	1,11
Debt ratio (%)	76,64	77,52

*Source: Cofarcir’s Annual Reports.*

### Technology and Innovation at Cofarcir

The pharmaceutical distribution sector, and particularly the company under analysis, has always been a pioneer in relation to the adoption of continuous IT developments. In the 80s, Cofarcir gave its pharmacies dataphones for the electronic transmission of medical orders, thus preceding the Internet and the official definition of e-business by several years. That initial step in the use of IT added to the robotic transformation of the company’s main warehouse, which took place in 1994 and which was an important step that allowed Cofarcir to fulfill two daily orders to each pharmacy in the region, thereby consolidating its position as regional leader. The automation of the other warehouse took place in 2005 and permitted the application of the most innovative technology in the sector worldwide.

The pharmaceutical sector in general, and the distribution of drugs in particular, is immersed in a continuous process of change in Spain. The worldwide crisis we have been confronting since 2008, which is more serious in this country, has resulted in constant decreases in the prices of medication, in subsidies for patients as regards acquiring them and in many other measures such as the promotion of generic products or the setting up of public pharmacy services inside hospitals. Government sanitary agencies meanwhile continue to push the ideal goal of total traceability of the whole medication chain, from the laboratory that produces it to the final patient who consumes the drug. This is an extremely difficult objective to achieve, and the most recent theoretical technologies are intended for this purpose. Thirdly, the great pressure that the huge wholesalers, which control the pharmaceutical distribution in other European countries, are exerting on Spain with the aim of changing the successful *Mediterranean model*. Finally, some large cooperatives in this sector have opted for expansion outside their natural territories.

Cofarcir has decided to confront these major challenges in two ways: firstly, it continues to rely on the current regional cooperative model, convinced that this is the optimal means to maintain the high standards of the *Mediterranean model*. To accomplish this goal Cofarcir has strengthened its ties with several other Spanish cooperatives that are united in this vision in order to create the group Unnefar, the fourth biggest of its kind in Spain. This group’s intention is to provide its members with the size needed for them to continue being a relevant piece of the system in defense of their principles. Secondly, Cofarcir wishes to improve the service it provides to its customers/owners, whilst enhancing its productivity. The company has therefore made the decision to renew the technology at its main warehouse in Ciudad Real. The automation level of this storehouse, although a pioneer when installed back in 1995, was becoming outdated and was virtually unable to keep pace with the new regulations commented on above. The company selected for this change in technology has again been Knapp, an Austrian company and a worldwide leader in this sector.

The core of the project is to install a chaotic storage system of 16 m-high towers – called OSR35b – in which the system automatically chooses the place where the goods are stored, allowing the separation of a good by its batch number and its expiration date. The user does not know where the products are kept inside the robot. They only ask the system for an amount of good and the system retrieves it to them, following certain premises (FIFO, for instance) while keeping track of which batch is sent to which pharmacy.

There are several options by which to ’feed’ that robot with the exact batch numbers and expiry dates. The best appear to rely on the information the laboratories send with the products when delivered to Cofarcir. However, this information is not always sent in the way-bill and worse, when it is, it is very difficult to introduce into the computer system as it is printed on paper. For the time being, Cofarcir will continue to use the AECOC standard which uses the GS1 bar-code system to describe much important information about the products delivered. This information is registered in Cofarcir with bar-code readers and special software developed internally, and is linked with the product income via radio-frequency devices when available.

The increase in productivity will principally occur in two ways. Firstly, although around 75% of the products will be dispatched automatically by new and existing robots (TDA, SDA, and HDA Knapp technologies), the remaining 30% will, owing to their fragile nature, great volume or high demand, have to be dispatched by hand. A new technology is now being used and improves the speed of manual dispatching by up to four times, while decreasing the error rate.

The last principal way in which the new technology will improve the service is with the use of a new robot – called OSR35ds – that will efficiently order the client packages into routes, thus saving the delivery van drivers’ time in addition to minimizing the possibility of mistakes as regards mixing up boxes. There is also a new positioning system, which has been developed by Cofarcir employees and which aims to use the Internet to inform clients where their order is and how long it will take them to receive it.

The total investment will be up to 6 million Euros. This amount includes not only the cost of the technology explained above but also the building of a new warehouse next the current one to contain it.

### Technology Entrepreneurship at Cofarcir and Its Main Effects

**FIGURE A1:**
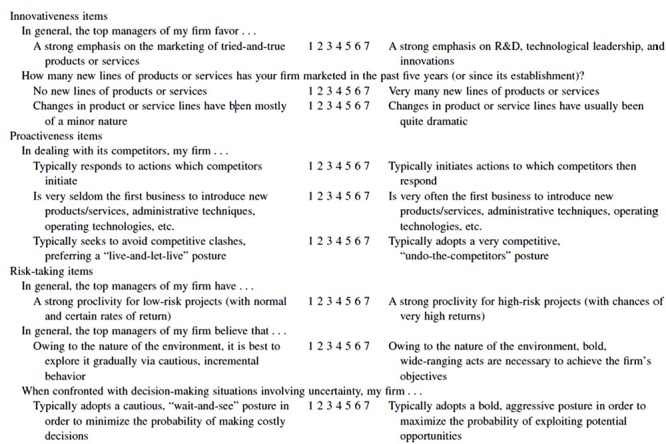
**The Covin and Slevin (1989) EO Scale.** *Source:* Covin and Wales (2012).

**FIGURE A2:**
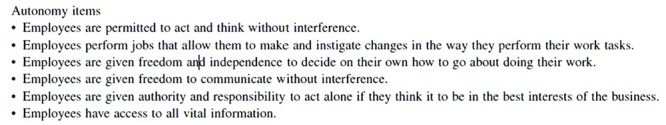
**Autonomy items of Hughes and Morgan (2007).** *Source:* Covin and Wales (2012).

We shall now present the main ideas extracted from the interviews (**Appendix Figures A1** and **A2**).

#### Entrepreneurial Orientation

Following the Covin and Slevin (1989) and the Hughes and Morgan (2007) scales we can consider that the company has an EO, i.e., it exhibits high levels of risk taking, innovativeness and proactiveness. In general terms, we can summarize that the organization has, over the last few years, marketed very many new lines of products and has placed a strong emphasis on R&D, technological leadership and innovation; in dealing with its competitors, it is very often the first business to introduce new techniques and operating technologies, adopting a very competitive posture; the company’s top managers have a strong proclivity for high-risk projects, consider that owing to the nature of the environment, wide-ranging acts are necessary to achieve the firm’s objectives and, when confronted with decision-making situations involving uncertainty, the firm typically adopts a bold, aggressive posture in order to maximize the probability of exploiting potential opportunities. However, upon considering the autonomy items proposed by Hughes and Morgan (2007), although employees are given freedom to communicate without interference and have access to all vital information, they are not given freedom to decide for themselves how to go about doing their work or to make and instigate changes as regards the way in which they perform their work tasks.

#### Organizational Effects

In order to analyze the new technologies’ organizational effects we consider Mintzberg’s (1979) structuring of organizations. IT influences the organizational structure via the company’s organizational design parameters: job design, design of the superstructure, design of the lateral linkages and design of the decision-making system.

In relation to job design, the IT in Cofarcir allows its workers to know how they are performing their jobs, thus allowing them to improve their skills in order to make their tasks more efficient. This may lead to increased motivation since the workers can evaluate their performance. It also makes the workers more multifunctional because they have to cope with the instructions, guides, checking, warnings, etc. implemented in their workloads, thus allowing them to learn new tasks faster. However, IT’s role as regards making work more creative is not so clear, since one of its main goals is standardization. In the design of the superstructure, there is no need to cut the number of managers required because, considering the firm’s innovative trajectory, it is already adjusted and is as flat as is necessary. In any case, IT helps workers to auto-organize their work whilst simultaneously providing management with powerful means of control, from the overall performance of the production system to the most detailed performance of an individual worker. This leads to a direct and efficient control of the company, eliminating intermediaries in the process.

In terms of jobs, owing to the considerable changes in the way that the work will be done, the company considers that there is no need to decrease the amount of warehouse workers. New specialized jobs such as an industrial engineer in charge of the new installation in order to control the new robot, and the reconversion of some warehouse workers into administrative workers are considered sufficient to offer better/new services to the customers. The automation of the firm has not therefore created or eliminated departments but has simply redistributed the employees to control, administration, maintenance and IT jobs since fewer workers are now directly needed in the production process. It is therefore necessary to make changes as regards the employees’ training not only in the mechanical area but also in those of management, maintaining, etc.

With regard to lateral linkages, tools like Business Intelligence Software provide the management with a better understanding of the process and allow them to determine the main variables that affect it, in addition to keeping track of the critical points involved. This leads to an improvement in the planning phase. Improved control is one of the main reasons for investing in IT. In the design of the decision-making system, IT eventually supports both centralization and decentralization. On one hand, as stated previously, managers need IT to be able to make decisions based on a better understanding of the development of the process. On the other hand, the exact information at the right time is vital for those processes that are directly related workers in order to prevent errors, correct deviations and assign more resources to those activities which are more demanding in each case.

Finally, the IT developed by Cofarcir has been strategically planned in such a way that it will have a lifecycle of about 20 years. This is not only a leading technology but also a change of paradigm with regard to the existing processes, and it will therefore have a long trajectory. Here we are referring to a vertical occupation of the space, the chaotic storage of goods, the intensive use of radiofrequency and the virtual elimination of the use of paper. The company is also preparing for new business lines. The technology installed should be improved in the future. In this respect, various complementary innovations have been planned, such as the installation of a massive new automatic dispatching robot (SDA), fed by the chaotic storing system currently installed (OSR), or the transformation of part of the manual dispatching of goods (picking) to robotic devices (currently in a prototype state).

This is the third technological “wave” developed by Cofarcir. The first took place 20 years ago, in the company’s main installations in Ciudad Real, and was the more disruptive innovation owing to the replacement of manual processes with robotic ones. The second technological change, which was similar to the first, took place in the Alcázar de San Juan installations. These prior experiences have permitted the company to maintain its leadership and to guarantee the success of the third “wave” development.

#### Company Strategy

Although part of the system is already in other companies, the use of the latest innovations has led the installer firm to consider Cofarcir as a model for other organizations (*show room*). The main reasons for the changes have been the obsolescence of some parts of the system and the need to adapt to the present requirements of pharmaceutical distribution. In this respect, the company is following a differentiation competitive strategy.

The entire process has been developed by Cofarcir itself, although its excellent relationship with other cooperatives in the sector has permitted it to share experiences and visit their installations. This benchmarking process is therefore a source of new ideas for the company, and these external partners are important sources of knowledge for Cofarcir, thus allowing it to keep up to date with the latest technological developments (Brettel and Cleven, 2012).

### Effects on Customers and Society

The present technological development at Cofarcir is having an important effect on pharmacies and, as a consequence, on customers and the population. All of them are able to receive two daily orders in no more than 3–4 h, so a *just in time* system has in fact been developed. Moreover, the improvements in the conservation and transportation of the medicines have led to a better delivery of the goods to the pharmacies. The social impact is also important, bearing in mind the special characteristics of the pharmaceutical sector. It is a sensitive sector for the population as all the activities are related to health.

All these characteristics make it possible to consider Cofarcir as a “market pioneer” or “first-mover.” The mechanism that produce a pioneering advantage is in some way able to slows the natural forces of competition, thus making it difficult for later entrants to attain a pioneer’s advantage. The process by which consumers learn about new products or services and form preferences for them play an important role in creating an advantage for pioneers. The first-mover can become strongly associated with the product category as a whole and, as a result, become the “standard” against which all later entrants are judged (Carpenter and Nakamoto, 1989).

## Conclusion

This study shows the organizational and social effects of IT entrepreneurship. We consider that it is a potential source of sustainable competitive advantage through the technical skills in technology and the ability to manage these new technologies. Technological entrepreneurs are more motivated than other entrepreneurs as regards starting projects and putting their innovative ideas into practice. They therefore tend to be driven by the need for achievement and self-realization and the desire to implement their projects. One area that is increasingly seeking ways in which to add value through innovation is that of the logistics function (Soosay and Hyland, 2004). Cofarcir is a pharmaceutical distribution leader that has been able to follow a successful technological strategy during its more than 75 years of life. The IT developed by Cofarcir is expected to have:

• Internal effects. Technology entrepreneurship may affect the organizational structure and the human resources strategy. There is general recognition that new technologies are changing the way in which people work. The main influences of the new IT is in relation to job design practices.
• External effects. Technology entrepreneurship influences the productivity of pharmacies, and consequently the population and the consumers.

The recent history of Cofarcir shows an important EO, this being principally a technology entrepreneurship which contributes toward improving its productivity. Here, we present a summary of the main improvements the company is expecting from its new technology development:

• The IT main objective is not the increase in the number of customers (the company provides to 350 pharmacies) but the productivity and customer service improvement. As entire categories of businesses move toward price, performance and tactical parity among products, customers are demanding new types of benefits that go beyond functional attributes, which are increasingly less differentiated (Singer, 2005). Marketplace success of an innovation is a combination of both consumer acceptance of the innovation and its appropriate roll-out in the marketplace which includes a well-executed marketing mix (Bagga et al., 2016).
• One consequence of IT is an improvement in the employees’ qualification (the Austrian Technology provider assumes the necessary training). In this respect, Cofarcir needs to reconvert some of its warehouse workers into administrative or commercial employees.
• The new IT increases the workers’ motivation and learning since they can evaluate their results.
• A better control of the whole process is going to be achieved because of the radiofrequency terminal technology used: what is the client’s order state, who dispatched what drug, when, at what rate... Minor delivery times due to better performance of the whole process (the boxes are pre-ordered per client and per delivery time by the system, the drugs dispatching time will decrease to four times).
• The company is going to avoid almost all dispatching errors due to barcode checking each product treated.
• Orders security will be improved due to the new strip surrounding the boxes before leaving the facilities.

Although, the managers of Cofarcir have a relevant amount of EO (this new technology is only one new phase in the innovation strategy), they maintain the traditional work system (*Mediterranean model*). The company also attempts to improve its knowledge by reaching cooperative agreements with other cooperatives. Cofarcir is therefore adapting its traditional strategy to changes in the environment. In this case we consider the managers’ attitudes to innovation to be particularly determinant. Fry (1987, p. 5) argued that:*“...if managers aren’t innovative, if they don’t provide the climate for creativity, if they can’t set aside their carefully laid plans to take advantage of a new opportunity, then intrapreneurs have little encouragement*.”

The research question we set out at the beginning of the paper concerned investigating “how” EO occurs in the company and “what” factors enable it. In this respect, this study makes several contributions to the literature on technological entrepreneurship. Our analysis of this company’s evolution has allowed us to extract the following key dimensions as determinants and consequences of its technology entrepreneurship (see **Table 3**).

**Table 3:** Characteristics and effects of Cofarcir’s technology entrepreneurship.

Entrepreneurial orientation	We have observed high levels of risk taking, innovativeness and proactiveness. Nevertheless, we have not detected autonomy for the employees,
Organizational effects	Multifunctional and more motivated employees Improvements to planning and control Training necessities, redistribution and specialization of employees The new technology’s long lifecycle
Company strategy	The company is a *show room* Differentiation Benchmarking
Social effects	Improvements in the delivery of medication to pharmacies and, consequently, to the population and the consumers

*Source: Authors.*

With regard to “how” and “what,” the results of the study therefore support the previous research developed by the authors referred to in the theory review. Risk taking, innovativeness and proactiveness are the entrepreneurial enablers for a company’s EO and for the successful result of the activities linked to it.

### Research Implications

This study has important implications for companies. Planning is necessary in order to obtain a successful strategy. This is typically analyzed in academic and practitioner communities as a fundamental managerial activity. Unfortunately, the contributions of planning are often difficult to quantify in practice (Segars and Grover, 1998) with an important gap between research and practice. It is generally accepted that one of the key factors for successful information systems planning and implementation is the close linkage with business strategy (Baets, 1992). Planning activities require substantial resources in terms of managerial time and budget. The process must therefore provide benefits beyond the resources needed to sustain it in order to make a positive contribution to organizational effectiveness (Segars and Grover, 1998). In this respect, Cofarcir’s managers have communicated a clear vision and plan to employees for a long term horizon, and this is one of the most important factors as regards explaining the company’s success and long life. We can also see how the entrepreneurs‘ innovating spirit does not expire with the creation of the project, and allows them to constantly redefine the business. For already established companies, this case illustrates the need for and usefulness of maintaining entrepreneurship tension throughout project development in order to favor the emergence of new and potentially complementary projects.

The improvement made to the whole process of product delivery is one of the most important results of Cofarcir’s IT entrepreneurship. Consumer service is now better in terms of: a decrease in the error rate, the speed of order dispatching, software to inform customers where their order is and how long it will take them to receive it, etc. As stated previously, Cofarcir can be considered a “market pioneer” but the consumers play an important role in making this pioneering advantage persistent. Our analysis therefore contains some suggestions as to how a successful relationship with consumers can be attained, i.e., achieving a competitive advantage by *influencing* consumer tastes rather than *responding* to them. Recent research in marketing and psychology suggests that consumers often use information already contained in existing product or service categories to learn about new ones. Consumers will learn about these innovations more quickly and with fewer mistakes if entrepreneurs delineate the appropriate information that should be transferred from each domain (Moreau et al., 2001). That is, it is not sufficient for a company to be able to follow a successful technological strategy, and the process of educating consumers about an innovation is necessary in order to influence how consumers will structure their representations of it. There are many ways in which consumers can learn about new products or services. The learning theories most frequently cited in marketing literature include category-based learning, analogies, and mental simulation (Hoeﬄer, 2003) and entrepreneurs should attain some knowledge of them in order to improve their relationships with consumers and obtain mutual benefits.

This article has several limitations that reveal possible avenues for further research. First, this study has applied an in-depth case study to a firm. This implies an understanding of a complex issue or object extending experience or adding strength to what is already known thanks to previous research. Case studies emphasize a detailed contextual analysis of a limited number of events or conditions and their relationships. However, the findings should therefore be simultaneously treated with caution and should be verified and validated in other firms and other sectors. The focus of this case study was limited to a few concepts (EO and organizational and social effects) which are important for technology entrepreneurship. Various future research opportunities may therefore be possible. In order to form a more comprehensive and integrative case study, some other variables that are possibly important to CE and technological entrepreneurship could also be included, such as the characteristics of cooperative agreement or the managers’ personalities. It will also be necessary to repeat this study within a few years in order to observe the long-term effects of this technology entrepreneurship and the innovations developed by the company.

## Author Contributions

RM: has developed the interviews with the company’s CEO; JSP: has made the theoretical review; IP: has made the empirical study; YS: has made the empirical study.

## Conflict of Interest Statement

The authors declare that the research was conducted in the absence of any commercial or financial relationships that could be construed as a potential conflict of interest.

## APPENDIX

### Interviews Items

#### Organizational Effects

• Do you think that IT has permitted workers to assume more self-control and their jobs to become more creative, motivating and multifunctional?
• Has IT led to a flatter organization, cutting down on both the number of managers required and the number of hierarchical levels (owing to the reduction in vertical specialization, teamwork, and the fact that IT can take on many of the managers’ communication, coordination and control functions, in addition to extending their control span)?
• Is planning affected by IT (does it improve analytical and design capacities, increase access to information, etc.)?
• Is control affected by IT (does it make it easier to access the results being controlled, calculate more sophisticated indicators, detect the reasons for deviations from the expected results, provide more effective communication systems, etc.)?.
• In this case, does IT support centralization in the decision-making process (giving top managers a greater control capacity) or decentralization (information becomes rapidly accessible at any point in the organization)?
• Has the new technology involved any changes in the company’s organizational structure?
• Has the new technology involved any changes in the company’s management?
• Does the new technology have a long lifecycle or is a rapid obsolescence expected?
• Is this the first technological innovation in the company? Does the firm have sufficient experience to ensure its success?
• Has the new technology generated new job and training necessities?

#### Company Strategy

• Does the new robot exist in any other enterprises? Do you consider Cofarcir to be a follower or a pioneering company? Why have the managers decided to invest in this new technology and what is the company’s competitive strategy?
• Has Cofarcir developed any type of alliances with other companies?

#### Effects on Customers and Society

• Has the entire process had any effect on customers and society? How will the pharmacies be affected by it?
